# O018: Evaluation of the efficacy of a novel hydrogen peroxide cleaner disinfectant concentrate

**DOI:** 10.1186/2047-2994-2-S1-O18

**Published:** 2013-06-20

**Authors:** PJ Teska, A Rushworth, M Theelen, J Jongsma

**Affiliations:** 1 Global Healthcare, Diversey Inc, Sturtevant, USA; 2 R&D, Diversey Inc, Amsterdam, The Netherlands

## Introduction

Multiple studies have shown that high-touch surfaces such as bed rails, the tops of bed tables, and supply carts play a role in the transmission of pathogens to susceptible patients and that there are gaps in current cleaning practices. While there are no validated studies proving the value of daily cleaning/disinfection in aiding infection prevention for patients, there are trends towards increased use of disinfectants in many areas of Healthcare. Hydrogen peroxide disinfectants are being increasingly evaluated in Europe due to their superior efficacy, safety, cleaning and sustainability profiles.

## Objectives

We tested a novel hydrogen peroxide cleaner disinfectant formula concentrate (hereafter the “formula”) against standardized EN test methods to determine if the formula was capable of meeting the European efficacy standards for Healthcare disinfection. We also performed cleaning tests to determine if the cleaning performance of the formula was consistent with that of a strong neutral cleaner.

## Results

Using EN-1276 and testing against *Pseudomonas aeruginosa*, *Enterococcus hirae*, *Staphylococcus aureas*, and *Escherichia coli*, the formula passed (>5 log reduction) in dirty conditions at 30 seconds at a 3.5% dilution and at 5 min at a 2.0% dilution.

Using EN-13697 and testing against *Pseudomonas aeruginosa*, *Enterococcus hirae*, *Staphylococcus aureas*, and *Escherichia coli*, the formula passed (>4 log reduction) in dirty conditions at 5 min at a 3.5% dilution.

Using EN-1650 and testing against *Candida albicans* and *Aspergillus niger*, the formula passed (>4 log reduction) in dirty conditions at 15 min at a 3.5% dilution.

Using a standardized Gardner Cleaning Test Method, the cleaning performance of the formula at 3.5% dilution was 67% soil removal. The neutral cleaner (Jontec 300) at 2% dilution was 72% and demi (demineralized) water was 54%. All tests were run in duplicate with results averaged.

## Conclusion

Our study showed that the formula meets the efficacy standards of multiple EN disinfectant test methods and provided comparable cleaning efficacy to a standard neutral cleaner. Thus this formula is a viable candidate for the European Healthcare market for customers desiring a hydrogen peroxide based one step cleaner disinfectant that is bactericidal and fungicidal.

## Disclosure of interest

P. Teska Employee of Diversey Inc, A. Rushworth Employee of Diversey Inc, M. Theelen Employee of Diversey Inc, J. Jongsma Employee of Diversey Inc.

